# Leukemic stem cell persistence in chronic myeloid leukemia patients in deep molecular response induced by tyrosine kinase inhibitors and the impact of therapy discontinuation

**DOI:** 10.18632/oncotarget.9182

**Published:** 2016-05-05

**Authors:** Jean Claude Chomel, Marie Laure Bonnet, Nathalie Sorel, Ivan Sloma, Annelise Bennaceur-Griscelli, Delphine Rea, Laurence Legros, Anne Marfaing-Koka, Jean-Henri Bourhis, Shanti Ame, Agnès Guerci-Bresler, Philippe Rousselot, Ali G. Turhan

**Affiliations:** ^1^ Laboratoire de Cancérologie Biologique, CHU de Poitiers, Poitiers, France; ^2^ INSERM U935, Poitiers, France; ^3^ Service d'Hématologie Biologique, Hôpital Paul Brousse, Villejuif, France; ^4^ INSERM U935, Villejuif, France; ^5^ Université Paris Sud, Le Kremlin-Bicêtre, France; ^6^ Service d'Hématologie Adulte, Hôpital Saint Louis, Paris, France; ^7^ INSERM UMRS-1160, IUH-Université Paris Diderot-Paris 7, Paris, France; ^8^ Service d'Hématologie Clinique, Hôpital l'Archet, Nice, France; ^9^ Service d'Hématologie Biologique, Hôpital Antoine Béclère, Clamart, France; ^10^ Service d'Hématologie-Greffe de Moelle, Institut Gustave Roussy, Villejuif, France; ^11^ Département d'Hématologie et Oncologie, Hôpitaux Universitaires de Strasbourg, Strasbourg, France; ^12^ Service d'Hématologie Clinique, CHU Brabois, Vandoeuvre les Nancy, France; ^13^ Service d'Hématologie et Oncologie, Centre Hospitalier de Versailles, Versailles, France; ^14^ EA4340, Université Versailles-Saint Quentin en Yvelines, Université Paris-Saclay, France; ^15^ Service d'Hématologie, Hôpital Bicêtre, Le Kremlin-Bicêtre, France

**Keywords:** chronic myeloid leukemia, leukemic stem cells, persistence, tyrosine kinase inhibitors, therapy discontinuation

## Abstract

During the last decade, the use of tyrosine kinase inhibitor (TKI) therapy has modified the natural history of chronic myeloid leukemia (CML) allowing an increase of the overall and disease-free survival, especially in patients in whom molecular residual disease becomes undetectable. However, it has been demonstrated that *BCR-ABL1-* expressing leukemic stem cells (LSCs) persist in patients in deep molecular response. It has also been shown that the discontinuation of Imatinib leads to a molecular relapse in the majority of cases. To determine a possible relationship between these two phenomena, we have evaluated by clonogenic and long-term culture initiating cell (LTC-IC) assays, the presence of *BCR-ABL1*-expressing LSCs in marrow samples from 21 patients in deep molecular response for three years after TKI therapy (mean duration seven years). LSCs were detected in 4/21 patients. Discontinuation of TKI therapy in 13/21 patients led to a rapid molecular relapse in five patients (4 without detectable LSCs and one with detectable LSCs). No relapse occurred in the eight patients still on TKI therapy, whether LSCs were detectable or not. Thus, this study demonstrates for the first time the *in vivo* efficiency of TKIs, both in the progenitor and the LSC compartments. It also confirms the persistence of leukemic stem cells in patients in deep molecular response, certainly at the origin of relapses. Finally, it emphasizes the difficulty of detecting residual LSCs due to their rarity and their low *BCR-ABL1* mRNA expression.

## INTRODUCTION

In the current era of tyrosine kinase inhibitor (TKI) therapies, chronic myeloid leukemia (CML), previously a deadly hematopoietic malignancy, has now become a truly chronic disease with low progression rates especially in patients receiving second-generation TKI as shown by trials comparing Nilotinib or Dasatinib to Imatinib [1, 2].

It has been clearly demonstrated that TKIs are efficient on most CD34+CD38+ and CD34+CD38- cells in CML patients [3, 4]. However, they appear unable to eradicate the most primitive quiescent leukemic stem cells (LSCs), as demonstrated by *in vitro* experiments [5–8] as well as clinical studies using patient-specific DNA PCR [9, 10]. In fact, although TKIs maintain their efficacy against the BCR-ABL tyrosine kinase, CML stem cells could escape from their oncogenic addiction [11, 12]. All these findings probably explain the fact that upon discontinuation of Imatinib in the context of deep molecular response, more than half of the patients relapse during the first six months [13–15]. Although the *in vitro* resistance of quiescent primitive stem cells to TKI was shown more than a decade ago [5], the *in vivo* study of quiescent primitive LSCs in CML patients is difficult as these cells are overgrown by normal stem cells [16], and the expression of *BCR-ABL1* mRNA is weak in the most primitive fraction [17, 18].

In previous work, including six patients in MR^4.5^ induced either by interferon-alpha alone, Imatinib following interferon treatment, or Dasatinib following Imatinib, we demonstrated the persistence of LSCs in all patients [19]. In that study, we designed a screening technique to measure the *BCR-ABL1* mRNA expression in individual and pooled hematopoietic colonies, allowing the evaluation of 220 colony-forming unit cells (CFU-Cs) per patient in clonogenic and long-term culture initiating cell (LTC-IC) assays. Our data were confirmed by Chu et al., who studied CD34+CD38+ and CD34+CD38- cells from CML patients in cytogenetic or molecular response [20]. These authors also showed that persistent leukemic stem cells or progenitors had long-term repopulating capacity in immunodeficient mice. In the present work, the presence of *BCR-ABL1*-expressing LSCs was investigated in CD34+ cells isolated from the bone marrow of 21 CML patients in sustained deep molecular response induced with a first-line Imatinib or Dasatinib. Findings were then related to the clinical outcome of the patients according to the presence of detectable LSCs and therapy discontinuation.

## RESULTS

### Analysis of hematopoietic progenitor and stem cell colonies

Twenty-one CML patients were included in the present study and had a bone marrow aspiration (Supplementary Table S1). At the time of the investigation, all patients were in persistent deep molecular response after TKI therapy. For some patients (P1-P13), the strategy was based on the analysis of an optimal number of 20 individual hematopoietic colonies and 20 pools of 10 colonies, from CFU-C and LTC-IC assays (when marrow CD34+ cells were in sufficient number). For the remaining patients (P14-P21), 40 individual hematopoietic colonies, from CFU-C and LTC-IC assays (when marrow CD34+ cells were in sufficient number) were analyzed. In the latter protocol, we did not pool the colonies in order not to dilute out *BCR-ABL1* mRNA, which we have found highly reduced in LSCs [18]. A total of 1073 individual and 3060 pooled colonies from CFU-C and LTC-IC assays were analyzed (Supplementary Table S1).

### Patients with detectable *BCR-ABL1*-expressing LSCs

Leukemic progenitors and/or stem cells were detected in four patients (Table 1). *BCR-ABL1*-expressing CFU-Cs were found in three patients (P2, P13, and P18). Given the small fraction of marrow analyzable, they demonstrated the presence of a significant amount of leukemic progenitors in the bone marrow. In one patient (P8), *BCR-ABL1* mRNA expression could not be observed in the CFU-C analyzes, but detected in the LTC-IC-derived progenitors. Patient 2 presented both leukemic CFU-Cs and LTC-IC-derived progeny in his bone marrow. It must be emphasized that the detection of *BCR-ABL1*-expressing LTC-ICs suggests the presence of a large number of LSCs in the bone marrow.

**Table 1:** LSCs detection and estimation of the amount of bone marrow CFU-Cs and LTC-ICs

A) CFU-Cs			
Patient	Detection of *BCR-ABL1*-expressing CFU-Cs		Estimation of the number of *BCR-ABL1*-expressing CFU-Cs (per ml of bone marrow)
P2	Yes	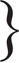	100- 360 CFU-Cs/ml
P13	Yes		
P18	Yes		
P8	No		

B) LTC-ICs			
Patient	Detection of *BCR-ABL1*-expressing LTC-ICs		Estimation of the number of *BCR-ABL1*-expressing LTC-ICs (per ml of bone marrow)
P2	Yes	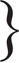	1000-4000 LTC-ICs/ml
P8	Yes		
P13	N/A		
P18	No		

LSCs: leukemic stem cells (expressing *BCR-ABL1* mRNA transcript), CFU-Cs: colony forming unit-cells, LTC-ICs: long term culture-initiating cells, N/A: not available (insufficient number of cells to initiate the test).

Tables 2A and 2B show the characteristics of the four patients in whom the presence of LSCs was demonstrated. Two patients (P13, P18) were still on Imatinib therapy and remained in molecular response. They have been treated for 9 and 12 years, respectively. Regarding patients in whom the TKI therapy was stopped, one patient (P8), presented a rapid molecular relapse two months after Imatinib discontinuation. In the current study, the molecular relapse was defined as a loss of the major molecular response (MMR) as previously established [21]. It should be emphasized that patient P8 has been retreated with Imatinib and regained a deep molecular response. The other patient (P2), with low Sokal Score, remained in MR^4.5^. Minor fluctuations in the *BCR-ABL1*/*ABL1* ratio were then observed in the patient's blood samples. The TKI duration in these patients was quite similar (7-8 years).

**Table 2:** Patients' outcome according to leukemic stem cell detection and TKI therapy

A) LSCs+ / TKI-ON				
Patient	Therapy	Sokal Score	Duration of TKI therapy (months)	Outcome
P13	Imatinib	High	108	Persistent deep MR (>MR^4^)
P18	Imatinib	Intermediate	144	Persistent deep MR (>MR^4^)

B) LSCs+ / TKI-OFF				
Patient	Therapy	Sokal Score	Duration of TKI therapy (months)	Outcome
P2	Imatinib	Low	89	Persistent deep MR (>MR^4^), fluctuation of the blood *BCR-ABL1*/*ABL1* ratio
P8	Imatinib	Intermediate	72	MMR loss, Imatinib retreatment, MR^4.5^ on Imatinib

C) LSCs- / TKI-ON				
Patient	Therapy	Sokal Score	Duration of TKI therapy (months)	Outcome
P1	Imatinib 800	High (AP)	84	Persistent deep MR (>MR^4^)
P5	Imatinib	N/A	144	Persistent deep MR (>MR^4^)
P10	Imatinib	High (AP)	150	Persistent deep MR (>MR^4^)
P12	Imatinib	Low	132	Persistent deep MR (>MR^4^)
P14	Imatinib	Intermediate	144	Persistent deep MR (>MR^4^)
P17	Imatinib	Intermediate	45	Persistent deep MR (>MR^4^)

D) LSCs- / TKI-OFF				
Patient	Therapy	Sokal Score	Duration of TKI therapy (months)	Outcome
P3	Imatinib	Low	92	Persistent deep MR (>MR^4^), fluctuation of the blood *BCR-ABL1*/*ABL1* ratio
P4	Dasatinib	High	19	Persistent deep MR (>MR^4^) until death from Alzheimer's disease
P6	Imatinib/ARA-C	Low	96	Persistent deep MR (>MR^4.5^), fluctuation of the blood *BCR-ABL1*/*ABL1* ratio
P7	Imatinib	Low	69	Persistent deep MR (>MR^4.5^), fluctuation of the blood *BCR-ABL1*/*ABL1* ratio
P9	Imatinib	Low	71	MMR loss, Imatinib retreatment, new Imatinib discontinuation, >MR^4^, fluctuation of the blood *BCR-ABL1*/*ABL1* ratio
P11	Imatinib	Low	81	Persistent deep MR (>MR^4.5^)
P15	Imatinib	High	84	Persistent deep MR (>MR^4^)
P16	Imatinib	High	28	MMR loss, Imatinib retreatment, MR^4.5^ on Imatinib
P19	Imatinib	Intermediate	65	MMR loss, Imatinib retreatment, MR^4.5^ on Imatinib
P20	Imatinib	Intermediate	47	Persistent deep MR (>MR^4^), fluctuation of the blood *BCR-ABL1*/*ABL1* ratio
P21	Imatinib	Intermediate	133	MMR loss, Imatinib retreatment, MR^4.5^ on Imatinib

TKI: tyrosine kinase inhibitor, LSCs: leukemic stem cells (expressing *BCR-ABL1* mRNA transcript), MR: molecular response, MMR: major molecular response, AP: accelerated phase, N/A: not available, ARA-C: cytarabine.

### Patients negative for LSC screening

In the majority of patients, neither *BCR-ABL1*-expressing CFU-C nor LTC-IC was detected despite a large number of hematopoietic colonies analyzed (average of 106 CFU-Cs and 75 LTC-ICs tested per patient). Table 2C shows the clinical characteristics of 6 such patients in whom the TKI therapy was maintained. These patients had either low (1), intermediate (n=2), or high (n=2) Sokal scores, and two patients were in accelerated phase at diagnosis (P1 and P10). The duration of Imatinib therapy varied between 3.5 and 12 years, and all patients remained in deep molecular response.

Table 2D provides the main features concerning patients in whom the TKI therapy was discontinued. Seven patients (P3, P4, P6, P7, P11, P15, P20) had no detectable LSC and did not relapse despite the discontinuation of TKI therapy (mean follow-up of 3.6 years). In this group of patients, the duration of TKI therapy varied between 19 months and eight years, and the Sokal score was high in two patients. Four patients (P9, P16, P19, P21) presented a molecular relapse after TKI discontinuation, despite the absence of detectable LSC. In these patients, the Sokal score was low in one, intermediate in two and high in one patient. The duration of TKI therapy was not shorter as compared to the previous group, and in one patient it was even the longest of the whole series with more than 11 years of TKI administration (P21). Furthermore, it must be pointed out that all patients who presented a molecular relapse after Imatinib cessation were retreated with Imatinib and achieved a molecular response again. Interestingly, patient P9 remained in deep molecular response after a second Imatinib withdrawal. In a significant number of patients in molecular response after TKI cessation, low levels of *BCR-ABL1* mRNA transcript were occasionally detected by qRT-PCR.

## DISCUSSION

**Figure 1:**
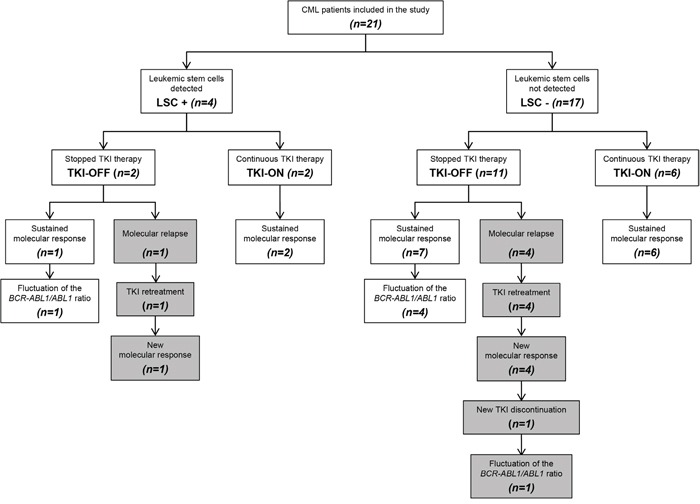
Relation between the detection of marrow leukemic stem cells and the clinical outcome according to therapy discontinuation

In this work, we used the most stringent *in vitro* LSC detection techniques to evaluate the effects of TKI as first-line therapies on the most primitive hematopoietic stem cell compartment. Data reported here demonstrate the *in vivo* efficiency of TKIs, both in the progenitor and the LSC compartments evaluated by LTC-IC assays. Indeed, only 4 out of 21 patients had detectable *BCR-ABL1*-expressing leukemic CFU-C and/or LTC-IC; a percentage quite low (19%) indicating the elimination of a large amount of leukemic stem cells by Imatinib or Dasatinib in the majority of CML patients (Figure 1). The present results suggest that an “*in vivo*” erosion of progenitor and LSC compartments is possible by TKI administered as first-line therapy.

Nevertheless, TKI discontinuation showed that four patients out of eleven with undetectable LSC had a molecular relapse after TKI withdrawal. Among the four patients with persistent LSCs, two had their therapy stopped, and one of them relapsed in two months (Figure 1), whereas the other remains in MR^4^. Regarding the relapse rate, this small series shows that a significant number of patients who stop their TKI lose the major molecular response, demonstrating the persistence of LSCs, detected only in some of them. All patients achieved a new molecular response when retreated. For patients still on TKI therapy with or without detectable LSC, it is not possible at this point to predict the potential outcome after TKI discontinuation. Consequently, our results suggest that the detectability of persisting CML marrow CFU-C or LTC-IC does not appear to be predictive of relapse after Imatinib cessation, and that other parameters could be involved, such as immunological surveillance.

The analysis of *BCR-ABL1* mRNA expression in the setting of individual LTC-IC-derived progenitors is especially challenging for several reasons. First, CML LSCs can be highly quiescent and, therefore, might not generate any detectable progeny in these assays. The second challenge is the rarity of this leukemic stem cell population underlining the methodological difficulty in identifying these cells in the bulk of the bone marrow niche, which protects them from TKI-induced death. Moreover, it has been shown that TKIs may relocate quiescent CML stem cells to the endosteal niche, making them difficult to be collected in the bone marrow aspiration [22, 23]. In addition, a potential toxicity of the TKI at the time of bone marrow aspirate need to be examined. Finally, for a still unknown reason, leukemic CFU-Cs and LTC-ICs isolated during TKI-induced remissions express very low amounts of *BCR-ABL1* mRNA [17, 18].

Our results raise the question of the detection of hematopoietic stem cells capable of reinitiating the disease upon TKI discontinuation since several such patients developed a molecular relapse without LSC detected in their bone marrow. This study clearly shows the need to improve the techniques of LSC detection for patients in deep molecular response, with the knowledge that the detection of LSCs is not correlated with disease relapse, especially in patients treated with interferon-alpha, as previously shown [19]. Finally, future studies will ask the question of purifying putative LSCs in the marrow samples using recently described leukemic stem cell markers such as CD26 [24, 25] or IL1RAP [26, 27].

Despite their efficacy in the inhibition of BCR-ABL kinase activity, TKIs appear to be ineffective against a small reservoir of CML stem cells not “oncogene-addicted” [11, 12]. The decrease of *BCR-ABL1* mRNA expression in CML progenitors and stem cells could represent a critical mechanism of LSC persistence [17, 18, 28]. The presence of a small contingent of CML stem cells in patients in deep molecular response might not necessarily cause the relapse of the disease. Minor fluctuations of *BCR*-*ABL1* mRNA transcript levels (<MMR) were observed in patients in sustained molecular response after TKI discontinuation. This phenomenon could be explained by the persistence of a residual pool of LSCs unable to initiate the leukemic process.

**Figure 2:**
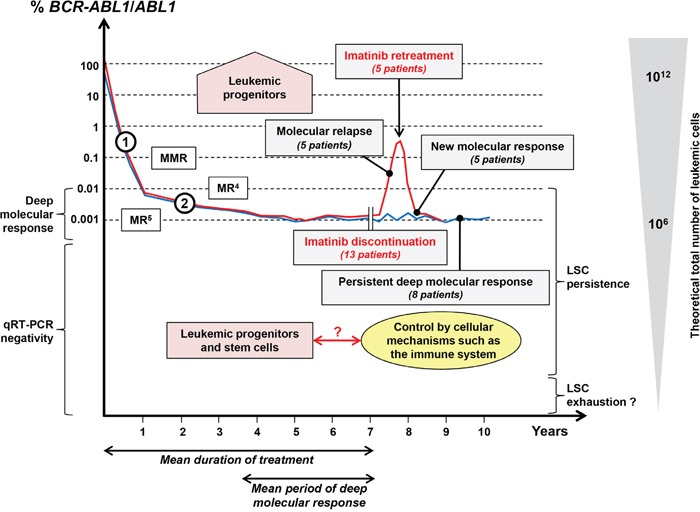
Molecular recurrence and sustained molecular response in CML patients after TKI discontinuation in the context of LSC persistence For patients included in the present study, a schematic blood *BCR-ABL1*/*ABL1* molecular monitoring is shown along with the theoretical total number of leukemic cells. Patients in molecular relapse after Imatinib discontinuation (red curve, 5 patients) provide clear evidence of the persistence of a pool of leukemic progenitors (detected or not in *in vitro* assays). All patients regained a deep molecular response after Imatinib retreatment. On the other hand, eight patients maintained a persistent deep molecular response after Imatinib cessation (blue curve) with a mean follow-up of 3.6 years. Fluctuations of the *BCR-ABL1*/*ABL1* ratio were observed in some of them. The theoretical persistence of LSCs in such a population (only detected in some patients) highlighted a potential role of the immune system in controlling the residual leukemic compartment. Too small an amount of LSCs could lead to the exhaustion of the LSC compartment. In this situation, the disease might be considered as “cured”. MMR: major molecular response; MR^4^ and MR^5^: deep molecular responses; LSCs: leukemic stem cells; Slopes (1) and (2) are indicative of the decline of mature and precursor/progenitor leukemic cells respectively.

Molecular recurrences in TKI discontinuation trials are undoubtedly the consequence of the persistence of a leukemic progenitor/stem cell reservoir (Figure 2). In this context, it is difficult to explain the maintenance of deep molecular remissions after Imatinib cessation. In theory, the persistence of LSCs could be at potential risk of relapse unless these cells are hierarchically different from one patient to another or unless they remain quiescent for prolonged periods of time. The classical immunological control concept of CML cell growth could be another factor of persistence. Recent work shows that KIR phenotype could be correlated and predictive of relapse after TKI discontinuation [29]. In addition, low amounts of NK cells were found to be associated with molecular relapse after Imatinib discontinuation [30]. These cells could represent an appropriate marker of immunosurveillance, which could discriminate CML patients who may safely stop the TKI therapy [31]. These data highlighted a potential role of the immune system in controlling the residual leukemic stem cell compartment. Moreover, in our previous work, larger amounts of CML stem cells were detected in patients who remained in deep molecular response after interferon-alpha withdrawal [19]. The pleiotropic activities of interferon-alpha, in particular its immune-modulating effects, could explain this phenomenon. Finally, an exhaustion of the progenitor/LSC reservoir might occur, after a slow erosion by TKI [32, 33]. In this particular situation, patients could be considered theoretically as “cured”.

From a clinical perspective, CML can be considered as an almost curable disease since a deep molecular response can be obtained by TKI therapy in a majority of patients. From a biological point of view, the persistence of LSCs, demonstrated by their *ex vivo* detection or the occurrence of molecular relapses following TKI discontinuation, remains a key issue that now deserves to be investigated using novel cellular and immunological approaches.

## MATERIALS AND METHODS

### Patients

A total of 21 CML patients (9 females, 12 males; mean age 49 years, range 25-86, referred as P1-P21) were included in the present study (Supplementary Table S1). They were diagnosed and treated in seven centers from the French intergroup of CML (Fi-LMC). At the onset, all patients were in the first chronic phase of the disease, except patients P1 and P10 who were diagnosed in accelerated phase. Sokal score was low in 7 patients, intermediate in 7 patients, and high in 6 patients. Molecular biology analyzes for the quantification of *BCR-ABL1* mRNA transcript were performed in each center and expressed on the international scale (IS). Deep molecular responses (MR^4^, MR^4.5^, and MR^5^) were assessed by the *BCR-ABL1*/*ABL1*^IS^ratio according to the standardized procedure [34]. At the time of the study, all patients were in MR^4.5^ except patient P18 (in MR^4^). Molecular responses were induced by Imatinib 400 mg daily in 18 patients, by Imatinib 400 to 600 mg daily for patient P18, by Imatinib 800 mg daily for patient P1, and by Dasatinib 100 mg/day for patient P4. For stem cell analysis, bone marrow aspirates were performed in all patients still receiving TKI therapy. The treatment was then discontinued in 13/21 patients. All patients provided informed consent for participation in this study, in accordance with the Declaration of Helsinki.

### Overview of the protocol

Bone marrow samples were collected and CD34+ cells purified. After performing a clonogenic assay, CD34+ cells were used in LTC-IC assays (Supplementary Figure S1). Hematopoietic colonies were plucked from clonogenic assays at day 0 and from LTC-IC-derived progenitors at week 5. Individual and pooled, or only individual colonies were tested for the presence of *BCR-ABL1* mRNA using reverse transcription-quantitative real-time PCR (qRT-PCR).

### *In vitro* hematopoietic progenitor and stem cell assays

Mononuclear cells were isolated from bone marrow samples on Histopaque density gradient separation (Sigma-Aldrich, St. Louis, MO). CD34+ cells were purified using immunomagnetic columns (Miltenyi Biotech, Paris, France). The purity of CD34+ cells, assayed by flow cytometry, varied between 90% and 95%. CFU-C assays (at day 0) were performed by plating 5,000 CD34+ cells on semisolid methylcellulose Methocult H4435 medium (StemCell Technologies, Vancouver, Canada). After 14 days of culture, hematopoietic colonies were enumerated, and a large fraction or the almost totality of CFU-Cs were plucked from methylcellulose and put (individually or by pools of 10 colonies) into RNA extraction buffer (Arcturus Bioscience Inc, Mountain View, CA). LTC-IC assays were carried out by seeding 40-60,000 CD34+ cells in MS5 stromal layers engineered to express HoxB4 protein, in order to amplify the number of leukemic LTC-IC, as previously described [19]. At week+5, cultures were sacrificed, and CFU-Cs originating from LTC-ICs were plucked, used for RNA extraction and analyzed for *BCR-ABL1* mRNA expression.

### Detection of *BCR-ABL1* mRNA on individual and pooled colonies

Total RNA was extracted from individual and pooled colonies using the PicoPure RNA isolation kit (Arcturus Bioscience Inc). RNA was reverse transcribed using the High Capacity cDNA Reverse Transcription Kit (Applied Biosystems, Foster City, CA). The presence of *BCR-ABL1* transcripts was analyzed by qRT-PCR as previously reported [19]. Each plate consisted of cDNA samples from hematopoietic colonies, positive and negative controls, and *ABL1* amplification was used to assess the presence of amplifiable cDNA. To be accurate, qRT-PCR experiments were conducted in duplicate and a minimum of 10,000 *ABL1* mRNA copies per well must be obtained. Finally, to be considered as *BCR-ABL1* positive, hematopoietic colony had to display valid and coherent *ABL1* and *BCR-ABL1* amplification curves in duplicate wells (difference in threshold cycles less than 1).

## SUPPLEMENTARY FIGURE AND TABLE




