# Supplementary material for: Leukemic stem cell persistence in chronic myeloid leukemia patients in deep molecular response induced by tyrosine kinase inhibitors and the impact of therapy discontinuation
Source: Oncotarget. 2016 May 5;7(23):35293–301. doi: 10.18632/oncotarget.9182 (PMC5085229; doi:10.18632/oncotarget.9182)
Supplement: Supplementary file 1 [file oncotarget-07-35293-s001.pdf]

Leukemic stem cell persistence in chronic myeloid leukemia patients in deep molecular response induced by tyrosine kinase inhibitors and the impact of therapy discontinuation

SUPPLEMENTARY FIGURE AND TABLE

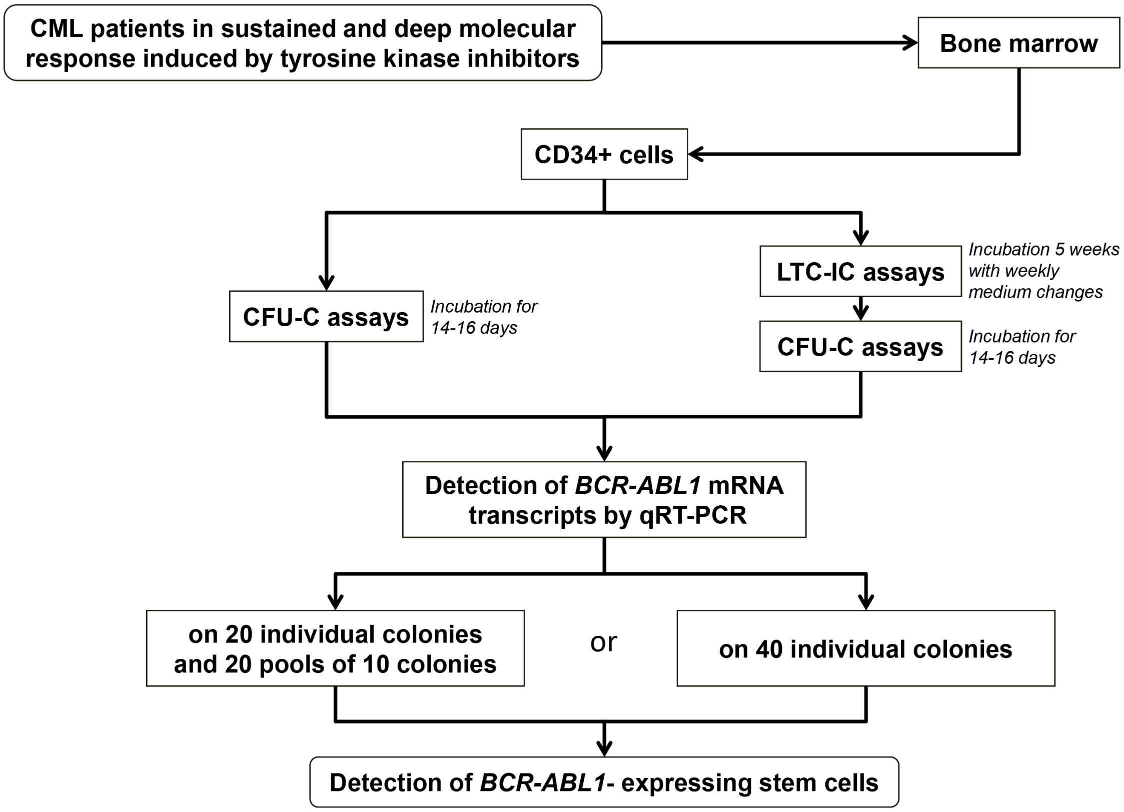

Supplementary Figure S1: Strategy used for the detection of *BCR-ABL1*-expressing leukemic stem cells.

Supplementary Table S1: Characteristics of patients included in the study and progenitor/LSC analysis on CFU-C and LTC-IC assays

See Supplementary File 1
