# Supplementary material for: Leukemic stem cell persistence in chronic myeloid leukemia patients in deep molecular response induced by tyrosine kinase inhibitors and the impact of therapy discontinuation
Source: Oncotarget. 2016 May 5;7(23):35293–301. doi: 10.18632/oncotarget.9182 (PMC5085229; doi:10.18632/oncotarget.9182)
Supplement: Supplementary file 2 [file oncotarget-07-35293-s002.docx]

| **Supplementary Table S1 : Characteristics of patients included in the study and progenitor/LSC analysis on CFU-C and LTC-IC assays.** | | | | | | | | | | | | | |  |  |  |
| --- | --- | --- | --- | --- | --- | --- | --- | --- | --- | --- | --- | --- | --- | --- | --- | --- |
|  |  |  |  |  |  |  |  |  |  | |  | |  |  |  |  |
| **Patient** | **Age/Sex** | **Sokal score** | **First-line therapy** | **Duration of TKI therapy (months)** | **Type of MR** | **Duration of MR (months)** | **TKI discontinuation** | **Outcome** | **CFU-C assays** | | **LTC-IC assays** | |  |  |  |  |
|  |  |  |  |  |  |  |  |  | Colonies analyzed | | Colonies analyzed | |  |  |  |  |
|  |  |  |  |  |  |  |  |  | individually | in pools  (10 colonies) | individually | in pools  (10 colonies) |  |  |  |  |
| **P1** | 63/M | High (AP) | Imatinib | 84 | MR^4.5^ | 56 | No | Persistent deep MR (>MR^4^) | 20 | 200 | 20 | 200 |  |  |  |  |
| **P2** | 48/M | Low | Imatinib | 89 | MR^4.5^ | 145 | Yes | Persistent deep MR (>MR^4^), fluctuation of the blood  *BCR-ABL1*/*ABL1* ratio | 30 | 200 | 20 | 200 |  |  |  |  |
| **P3** | 41/M | Low | Imatinib | 92 | MR^4.5^ | 34 | Yes | Persistent deep MR (>MR^4^), fluctuation of the blood  *BCR-ABL1*/*ABL1* ratio | 20 | 10 | *(1)* | |  |  |  |  |
| **P4** | 86/F | High | Dasatinib | 19 | MR^4.5^ | 18 | Yes | Persistent deep MR (>MR^4^) until death  from Alzheimer's disease | 20 | 200 | 40 | 280 |  |  |  |  |
| **P5** | 47/F | N/A | Imatinib | 144 | MR^4.5^ | 84 | No | Persistent deep MR (>MR^4^) | 20 |  | 20 |  |  |  |  |  |
| **P6** | 27/F | Low | Imatinib/ARA-C | 96 | MR^4.5^ | 52 | Yes | Persistent deep MR (>MR^4.5^), fluctuation of the blood  *BCR-ABL1/ABL1* ratio | 19 | 120 | 20 | 200 |  |  |  |  |
| **P7** | 25/M | Low | Imatinib | 69 | MR^4.5^ | 48 | Yes | Persistent deep MR (>MR^4.5^), fluctuation of the blood  *BCR-ABL1/ABL1* ratio | 20 | 200 | 40 |  |  |  |  |  |
| **P8** | 35/F | Intermediate | imatinib | 72 | MR^4.5^ | 29 | Yes / IM-R | MMR loss, Imatinib retreatment,  MR^4.5^ on Imatinib | 20 | 200 | 20 | 200 |  |  |  |  |
| **P9** | 40/M | Low | Imatinib | 71 | MR^4.5^ | 33 | Yes / IM-R / Yes | MMR loss, Imatinib retreatment, new Imatinib discontinuation,  >MR^4^, fluctuation of the blood *BCR-ABL1*/*ABL1* ratio | 20 | 200 | 20 | 200 |  |  |  |  |
| **P10** | 50/F | High (AP) | Imatinib | 150 | MR^4.5^ | 21 | No | Persistent deep MR (>MR^4^) | 19 |  | 5 |  |  |  |  |  |
| **P11** | 33/M | Low | Imatinib | 81 | MR^4.5^ | 36 | Yes | Persistent deep MR (>MR^4.5^) | 20 | 200 | *(1)* | |  |  |  |  |
| **P12** | 67/M | Low | Imatinib | 132 | MR^4.5^ | 74 | No | Persistent deep MR (>MR^4^) | 20 | 200 | *(1)* | |  |  |  |  |
| **P13** | 59/M | High | imatinib | 108 | MR^4.5^ | 40 | No | Persistent deep MR (>MR^4^) | 20 | 50 | *(1)* | |  |  |  |  |
| **P14** | 67/M | Intermediate | Imatinib | 144 | MR^4.5^ | 25 | No | Persistent deep MR (>MR^4^) | *40* |  | 40 |  |  |  |  |  |
| **P15** | 32/M | High | Imatinib | 84 | MR^4.5^ | 50 | Yes | Persistent deep MR (>MR^4)^ | 40 |  | *(1)* | |  |  |  |  |
| **P16** | 35/M | High | Imatinib | 28 | MR^4.5^ | 54 | Yes / IM-R | MMR loss, Imatinib retreatment,  MR^4.5^ on Imatinib | 40 |  | 40 |  |  |  |  |  |
| **P17** | 54/M | Intermediate | Imatinib | 45 | MR^4.5^ | 3 | No | Persistent deep MR (>MR^4^) | 40 |  | 40 |  |  |  |  |  |
| **P18** | 61/F | Intermediate | Imatinib | 144 | MR^4^ | 30 | No | Persistent deep MR (>MR^4^) | 40 |  | *40* |  |  |  |  |  |
| **P19** | 77/F | Intermediate | Imatinib | 65 | MR^4.5^ | 25 | Yes / IM-R | MMR loss, Imatinib retreatment,  MR^4.5^ on Imatinib | 40 |  | *40* |  |  |  |  |  |
| **P20** | 47/F | Intermediate | Imatinib | 47 | MR^4.5^ | 27 | Yes | Persistent deep MR (>MR^4^), fluctuation of the blood  *BCR-ABL1/ABL1* ratio | 40 |  | *40* |  |  |  |  |  |
| **P21** | 30/F | Intermediate | Imatinib | 133 | MR^4.5^ | 88 | Yes / IM-R | MMR loss, Imatinib retreatment,  MR^4.5^ on Imatinib | 40 |  | 40 |  |  |  |  |  |
|  |  |  |  |  |  |  |  |  |  |  |  |  |  |  |  |  |
| LSCs: leukemic stem cells (expressing *BCR-ABL1* mRNA transcript), CFU-Cs: colony forming unit-cells, LTC-ICs: long term culture-initiating cells, AP: accelerated phase, N/A: not available, ARA-C: cytarabine, TKI: tyrosine kinase inhibitors, MR: molecular response, MMR: major molecular response, IM-R: Imatinib retreatment, (1): insufficient number of CD34+ cells to initiate *in vitro* assays. | | | | | | | | | | | | |  |  |  |  |
